# Supplementary material for: Understanding age at menarche: Environmental and demographic influences over a quarter century in India
Source: PLOS Glob Public Health. 2025 Sep 17;5(9):e0005133. doi: 10.1371/journal.pgph.0005133 (PMC12443301; doi:10.1371/journal.pgph.0005133)
Supplement: S1 Text — (DOCX) [file pgph.0005133.s001.docx]

Table 1: Diagnostic Tests for Regression Assumptions in the 1992 and 2019 Models Using White’s Test and Cameron & Trivedi’s IM Test.

| **Test Type** | **Result** | **p-value** |
| --- | --- | --- |
| White’s test | chi²(83) = 474.83 (1992), 534.98 (2019) | **p = 0.0000** |
| Skewness test | Significant | **p = 0.0000** |
| Kurtosis test | Significant | **p = 0.0000** |
| Overall IM test | Total chi² = very high | **p = 0.0000** |
