# Supplementary material for: Understanding age at menarche: Environmental and demographic influences over a quarter century in India
Source: PLOS Glob Public Health. 2025 Sep 17;5(9):e0005133. doi: 10.1371/journal.pgph.0005133 (PMC12443301; doi:10.1371/journal.pgph.0005133)
Supplement: Attachment — Submitted filename: India-Global.docx [file pgph.0005133.s002.docx]

Thank you for the opportunity to review the manuscript entitled: Shifts in Menarche Age: Environmental and Demographic Influences Over a Quarter 2 Century in India. The study is relevant and contributes to the field.

This manuscript provides valuable insights into the complex drivers of menarche timing in India. Addressing the above suggestions, particularly deepening the discussion of Maharashtra’s outlier status, justifying methodological choices, and strengthening policy linkages, will enhance its impact for publication in PLOS Global Public Health.

1. The first sentence of the Introduction section needs to be rewritten.
2. Reference 6, which points to the trend of menarche age in China, is too old and unsuitable for mentioning and comparing. Please find newer studies and refer to them.
3. Please write the Introduction section in 4 or 5 separate paragraphs, but link the contents with related sentences.

**Results section:**

Simplify the data analysis subsection by condensing technical details (e.g., heteroscedasticity correction) into a separate supplementary section.

Ensure figures (e.g., bar charts for state-wise trends) are clearly labeled and visually accessible. For example, Figure 1’s y-axis should explicitly state "Mean Age (Years)" to avoid ambiguity.

**Discussion**

- provide specific socioeconomic or dietary data (e.g., NFHS reports, state-level nutrition surveys) to contextualize why Maharashtra diverges from national trends.
- Discuss potential biological or environmental mechanisms (e.g., heat stress adaptation, hormonal changes) for the shift from a negative (1992) to positive (2019) temperature effect.
- Clarify why higher education correlates with earlier menarche despite global trends linking education to delayed menarche. Consider regional factors like urban-rural disparities in educational quality or health access.

**Statistical Analysis**

- Justify the use of OLS over mixed-effects or longitudinal models, given the cross-sectional, time-segmented design.
- Address limitations of using only two time points (1992 and 2019) by discussing whether intermediate trends (e.g., 2005-06 NFHS data) were explored or could refine results.

#### ****Limitations****

- Expand on potential recall bias mitigation strategies (e.g., DHS survey protocols, validation studies).
- Acknowledge that excluding states due to data gaps (e.g., northern regions like Punjab) may limit national representativeness.

#### ****Policy Implications****

- Specify how public health programs in Maharashtra could address delayed menarche (e.g., nutrition supplementation, heat-resilient healthcare infrastructure).
- Link findings to India’s climate action plans (e.g., National Action Plan on Climate Change) to emphasize urgency.
- Revise fragmented sentences (e.g., "We used India's Demographic and Health Survey dataset. (DHS) contains...") and reduce redundancy in the methodology.
- Verify that all in-text citations (e.g., [20] for latitude/longitude sources) are correctly formatted and accessible.
