# Supplementary material for: Understanding age at menarche: Environmental and demographic influences over a quarter century in India
Source: PLOS Glob Public Health. 2025 Sep 17;5(9):e0005133. doi: 10.1371/journal.pgph.0005133 (PMC12443301; doi:10.1371/journal.pgph.0005133)
Supplement: Attachment — Submitted filename: Response to reviewers.docx [file pgph.0005133.s004.docx]

**Reviewer #1 Comments:**

**Comment 1:** The main factors affecting Menarche:

1. Heredity
2. Hormones
3. Amount of fat in the body
4. Health condition
5. Physical exercise

None of these factors were used as a control for climate factors. If only demographic variables are used as controls, it is not appropriate to see the role of climate.

**Response 1:** Thank you for your insightful comment. We fully acknowledge that biological determinants such as heredity, hormonal profile, and physical activity are critical factors influencing the age of menarche. However, due to the nature of the datasets used—namely, the Demographic and Health Surveys (DHS) and NASA POWER climate data—such information was not appropriately available. Consequently, we adjusted for a range of demographic factors—including residence, education, religion, and ethnicity—and focused on specific environmental indicators such as annual mean temperature and specific humidity from the NASA POWER dataset. These climate variables were used to represent environmental exposures that may influence biological processes related to menarche timing. This limitation has now been explicitly stated in the revised Limitations sections of the manuscript. We have also noted this gap as a direction for future research using richer clinical or biomarker-linked datasets.

**Comment 2:** The DHS data uses a cross-sectional research design, so it is not possible to see the effect. The purpose of the analysis is to see the relationship between the dependent variable and the independent variable

**Response 2:** We have revised the study design section accordingly. Thank you.
**Comment 3:** There is no explanation of the data management process for data coming from several sources.

**Response 3:** Thank you for this observation. The Data Analysis section has been substantially revised to provide a clearer explanation of the analytical framework and rationale for the statistical techniques employed in the study.

**Comment 4:** Is the availability of regional data in climate data the same as DHS data? Explain

**Response 4:** This has been addressed in the Data Preparation and Integration subsection. We clarify that while DHS data are structured by administrative state units, NASA POWER climate data are gridded. To ensure compatibility, DHS cluster GPS coordinates were used to extract and aggregate climate data at the state level, thereby harmonizing both datasets spatially.

**Comment 5**: What is the timing of climate observations and individual menarche times? Has it been adjusted for each individual to provide an explanation of the relationship between menarche and climate?

**Response 5:** Thank you for raising this key point. We have revised the Data Preparation and Integration subsection to explain that the year of menarche for each individual was estimated by subtracting their reported age at menarche from their age at interview. This derived year was then matched with the corresponding year’s climate data to ensure temporal alignment between climate exposure and menarche timing.

**Reviewer #2 Comments:**

**Comment 1:** The first sentence of the Introduction section needs to be rewritten.
**Response 1:** The first sentence of the introduction section has been revised. Thanks.

**Comment 2:** Reference 6, which points to the trend of menarche age in China, is too old and unsuitable for mentioning and comparing. Please find newer studies and refer to them.
**Response 2:** Upon review, we found that the referenced line, which cited an outdated study on menarche trends in China, may appear unsuitable. As its removal does not affect the overall flow or integrity of the introduction, we have decided to omit it. Thank you for your valuable feedback.

**Comment 3:** Please write the Introduction section in 4 or 5 separate paragraphs, but link the contents with related sentences.

**Response 3:** We have revised the introduction section and separated the paragraph into 5 separate paragraphs. Thank you.

**Comment 4:** Simplify the data analysis subsection by condensing technical details (e.g., heteroscedasticity correction) into a separate supplementary section.
Response 4: Additional supplementary added, and the data analysis section has been revised accordingly. Thank you.

**Comment 5:** Ensure figures (e.g., bar charts for state-wise trends) are clearly labeled and visually accessible. For example, Figure 1’s y-axis should explicitly state "Mean Age (Years)" to avoid ambiguity.

**Response 5:** Thank you for your feedback. We have revised all the visual figures and corrected the errors accordingly

**Comment 6:** Discussion

- Provide specific socioeconomic or dietary data (e.g., NFHS reports, state-level nutrition surveys) to contextualize why Maharashtra diverges from national trends.
- Discuss potential biological or environmental mechanisms (e.g., heat stress adaptation, hormonal changes) for the shift from a negative (1992) to positive (2019) temperature effect.
- Clarify why higher education correlates with earlier menarche despite global trends linking education to delayed menarche. Consider regional factors like urban-rural disparities in educational quality or health access.

**Response 6**: The discussion section has been revised according to the suggestion. Thank you.

**Comment 7:** Statistical Analysis

- Justify the use of OLS over mixed-effects or longitudinal models, given the cross-sectional, time-segmented design.
- Address limitations of using only two time points (1992 and 2019) by discussing whether intermediate trends (e.g., 2005-06 NFHS data) were explored or could refine results.
  Limitations
- Expand on potential recall bias mitigation strategies (e.g., DHS survey protocols, validation studies).
- Acknowledge that excluding states due to data gaps (e.g., northern regions like Punjab) may limit national representativeness.

**Response 7:** The Data Analysis section has been revised accordingly to address each of the queries. Thank you.

**Comment 8:** Policy Implications

- Specify how public health programs in Maharashtra could address delayed menarche (e.g., nutrition supplementation, heat-resilient healthcare infrastructure).
- Link findings to India’s climate action plans (e.g., National Action Plan on Climate Change) to emphasize urgency.
- Revise fragmented sentences (e.g., "We used India's Demographic and Health Survey dataset. (DHS) contains...") and reduce redundancy in the methodology.
- Verify that all in-text citations (e.g., [20] for latitude/longitude sources) are correctly formatted and accessible.

**Response 8**: All the comments have been addressed and revised within the discussion section. Part 3 comment has been addressed by revising the study design section. Also, citations have been checked and they are easily accessible. Thank you.
