# Supplementary material for: Understanding age at menarche: Environmental and demographic influences over a quarter century in India
Source: PLOS Glob Public Health. 2025 Sep 17;5(9):e0005133. doi: 10.1371/journal.pgph.0005133 (PMC12443301; doi:10.1371/journal.pgph.0005133)
Supplement: Attachment — Submitted filename: Second Response to reviewers.docx [file pgph.0005133.s005.docx]

**Reviewer #1 comments:**

**Comment 1:** In the introduction, the issue of menarche can be clarified. What is the ideal age for menarche? What are the effects of early or late menarche?

**Response 1:** Thank you for your comment. We have clarified it in the first paragraph of the introduction section. Thanks.

**Comment 2:** The term “trend” cannot be used because there are only two data points.

**Response 2:** Thank you for pointing this out. We agree that the use of the term “trend” is not appropriate when comparing only two time points. We have carefully revised the manuscript and replaced the word “trend” with more accurate terms, such as “change,” throughout, to ensure consistency and clarity.

**Comment 3:** The use of climate data that does not align with the age of menarche, for example, a respondent who was 15 years old during the 1992 survey and reported menarche at age 13 should have used climate data from 1990 (two years prior).

**Response 3:** Thank you for your important comment. We have added the response in the Data Preparation and Integration section. Thanks.

**Comment 4:** Suggestion: if the argument is that climate conditions have not changed significantly, then both climate datasets should be averaged. Additionally, the climate data used for analysis is only from 2019, while the climate data uses the average of data from 1992 and 2019.

**Response 4:** We have added the response in the Data Preparation and Integration section. Thank you.

**Comment 5:** What is the basis for using the religion indicator as a predictor? Explain with appropriate literature support.

**Response 5:** We appreciate the reviewer’s insightful question. Religion was included as a predictor because it often reflects underlying sociocultural norms, gender roles, dietary practices, and health-seeking behaviors, all of which may influence pubertal timing, including age at menarche. Prior studies have shown that religious affiliation can indirectly impact reproductive health outcomes by shaping attitudes toward education, nutrition, body image, and access to healthcare. We have now clarified this in the method of the manuscript (independent variable section) and added relevant references to support the inclusion of religion as a contextual demographic variable.

**Comment 6:** The conclusion is unclear. If climate plays a role in menarche, what solutions can be implemented by the community? Are there any interventions that can be carried out or adaptations that the community can make?

**Response 6:** We have revised the conclusion section. Thank you.

**Comment 7:** Re-analyze so that the theory is more consistent with the data used. Another thing to consider after changing the analysis is the title of the manuscript.

**Response 7:** Thanks for your suggestion. Upon reviewing, we have changed the title. Thanks.
