# Supplementary material for: Understanding age at menarche: Environmental and demographic influences over a quarter century in India
Source: PLOS Glob Public Health. 2025 Sep 17;5(9):e0005133. doi: 10.1371/journal.pgph.0005133 (PMC12443301; doi:10.1371/journal.pgph.0005133)
Supplement: Attachment — Submitted filename: Response to reviewers_R3.docx [file pgph.0005133.s006.docx]

**Reviewer #1 comments**

**Comment 1:** Add a theory that explains
a) How climate affects menarche
b) at what age menarche should occur, and the effects of menarche occurring too early or too late.

**Response 1:** The introduction section has been revised accordingly. Thanks.

**Comment 2:** Check line 279, is the sentence complete?

**Response 2:** The line has been revised. Thanks.

**Comment 3:** Add limitations regarding the status of climate data that is not ideal. The years of climate data collection and menarche are not identical.

**Response 3:** We have addressed this in the limitations section. Thanks.

**Comment 4:** Explain the role of religion in differences in menarche. Do religious rules cause these differences?

**Response 4:** We have added a paragraph in the Introduction section explaining how religion can affect menarche, and the revision has been incorporated into the paper. Thank you.

**Reviewer #2 comments**

**Comment 1:** In the Abstract: Abbreviate the methods section (e.g., "cross-sectional analysis of DHS and NASA data" is enough).

**Response 1:** We have revised the abstract method section according to the suggestion. Thanks.

**Comment 2:** In the introduction section, Condense the literature review; certain paragraphs (e.g., comparisons of caste) might be shortened.

**Response 2:** The introduction section has been revised and condensed. Thanks.

**Comment 3:** In the Methodology: Self-Report Bias, although mentioned, the effect of recall bias on menarche age (retrospective data) requires more in-depth discussion.

**Response 3:** Detailed information has been provided in the Inclusion and Exclusion Criteria section. Thanks.

**Comment 4:** Climate Data Granularity: Annual averages can hide seasonal and climatic extremes. Explain why multi-year averages were not used.

**Response 4:** Detailed information has been provided in the Inclusion and Exclusion Criteria section. Thank you.

**Comment 5:** Missing Variables: Lack of biological markers (hormones, BMI) is a significant limitation. Propose proxy variables (e.g., DHS wealth index for nutrition).

**Response 5:** Thank you for the comment. While BMI is a valuable biological marker, harmonized BMI data were not consistently available across the DHS 1992–93 and 2019–21 surveys. As this study relies on secondary data, such limitations in variable availability are inherent. Therefore, we used proxy indicators such as education, place of residence, and ethnicity, which are well-established correlates of nutritional and health status in India. Moreover, we have revised our limitation section for further clarification. Thanks.

**Comment 6:** In the Statistical presentation: Tables/Figures: Be consistent in formatting (e.g., confidence intervals in Table 2). Report p-values with coefficients.

**Response 6:** Thank you for addressing this. All p-value indications have been maintained consistently, and p-values are reported alongside the coefficients. Thank you

**Comment 7:** Supplemental Data: The test for heteroscedasticity (Supplement 1) needs to be summarized in the main text.

**Response 7:** This has been addressed in Regression Modeling and Inference section. Thanks.

**Comment 8:** In the findings: Education Paradox: The association of earlier menarche with higher education is contrary to international trends. Discuss possible mechanisms (e.g., school lunch programs vs. stress/physical activity).

**Response 8:** This has been revised in discussion section. Thanks.

**Comment 9:** Avoid repetitive statements (for example, Maharashtra's anomaly is stated more than once).

**Response 9:** We have revised this. Thanks.

**Comment 10:** Maharashtra's Anomaly: Speculate as to why this state differs (e.g., local droughts, migration patterns).

**Response 10:** This has been revised in the discussion section. Thanks.

**Comment 11:** In the discussion section, Climate Mechanisms: Explain how humidity/temperature physiologically impact puberty (e.g., hydration, thermoregulation stress).

**Response 11:** The discussion section has been revised accordingly. Thanks.

**Comment 12:** Elaborate on mechanisms underlying crucial findings (e.g., Maharashtra's trend).

**Response 12:** The discussion section has been revised accordingly. Thanks.

**Comment 13:** Discussed limitations more critically (e.g., biological confounders).

**Response 13:** Thank you for the insightful suggestion. We have expanded the limitations section to more critically discuss the absence of biological and physiological confounders, such as BMI, hormonal levels, and hereditary factors, which are known to influence menarche timing. We acknowledged this as a constraint of using secondary datasets like DHS and NASA POWER and emphasized the need for future biomarker-based studies to address this gap. Thanks.

**Comment 14:** Ethical and Generalizability Notes: State Selection Bias: Excluded states (e.g., Punjab, Jammu & Kashmir) can distort findings. Address regional representativeness.

**Response 14:** We appreciate this important observation. We have added language in the limitations section to acknowledge that the exclusion of certain states due to incomplete or inconsistent data may introduce selection bias and limit the generalizability of the findings. This limitation has been discussed explicitly to reflect the study's regional scope.

**Comment 15:** Open Data: Indicate whether raw/processed data are made available (e.g., through repositories such as Figshare).

**Response 15:** Thank you for the suggestion. We have clarified in the Availability of Data and Materials section that the raw datasets are openly available through the DHS Program and NASA POWER project.
